# COGNITIVE BEHAVIORAL AND VIRTUAL REALITY TREATMENTS FOR INSOMNIA (CBTI AND IVR): OFF-LABEL IMPACT ON COGNITION

**DOI:** 10.1093/geroni/igad104.1205

**Published:** 2023-12-21

**Authors:** Christina McCrae, Melanie Stearns, Ashley Curtis

**Affiliations:** University of South Florida, Tampa, Florida, United States; University of South Florida, Tampa, Florida, United States; University of South Florida, Tampa, Florida, United States

## Abstract

Chronic insomnia (CI) is common in mid-to-late life and associated with hyperarousal and impaired cognition and mood. CBTi improves sleep and may also improve cognition, arousal and mood but evidence for these latter outcomes is limited. We examined these outcomes across 3 studies with different delivery platforms (telehealth, digital, VR), dosages (2 sessions, 4 sessions), and (primarily) mid-to-older CI populations (fibromyalgia, dementia caregivers). Study 1 compared 8-week CBTi vs sleep hygiene in women with fibromyalgia (n=43, Mage=44.45). Study 2 piloted 4-session web-based CBTi in caregivers (n=5, Mage=62.40). Study 3 piloted 4-session iVR (n=18, Mage=36.45). Participants completed 2-week daily diaries pre/post treatment (sleep onset latency-SOL; wake after sleep onset-WASO; total sleep time-TST) and Insomnia Severity Index-ISI. Other measures included: study 1(arousal/heart rate variability-RMSDNN), studies 1 and 3(Wisconsin Card Sort Test-WCST, Stroop), study 2(Cognitive Failures Questionnaire-CFQ, Beck Depression Inventory-BDI-II, Perceived Stress Scale). Group x time interactions (study 1) and within-group pre/post differences were examined. CBTi improved sleep across studies (ps<.05). Study 1 found and study 3 trended toward improved cognitive flexibility (WCST %perseverative errors-t(14)=2.65, p=.019 and t(10)=1.76, p=.055, respectively). Study 1 found improved attention and processing speed [Stroop reaction time(RT)-congruent trials-t(14)=2.59, p=.023], inhibition (Stroop RT-incongruent trials-t(14)=2.43, p=.031), and arousal [increased RMSDNN; F(1,41)=4.87, p=.03]. Study 2 found improved subjective cognition-CFQ (t(4)=2.44, p=.04), arousal-RMSDNN (t(4)=2.09, p=.05), and depression-BDI-II (t(4)=2.89, p=.02). CBTi improved sleep, cognition, arousal and mood in mid-to-older CI populations. Research using randomization, active controls, and follow-ups is needed to delineate temporality and explore sleep’s mechanistic contribution to cognition and other “off-label” outcomes.

